# Geographic Variation in Neighborhood Stress, Discrimination, and Cognition in Black Midlife and Older Adults

**DOI:** 10.1093/geroni/igaf122.894

**Published:** 2025-12-31

**Authors:** Amy Thierry, Kyler Sherman-Wilkins

**Affiliations:** Xavier University of Louisiana, New Orleans, Louisiana, United States; Missouri State University, Springfield, Missouri, United States

## Abstract

**Background:**

Black Americans have twice the risk of developing Alzheimer’s disease (AD) than white adults. Historical racial inequities in neighborhood environments and experiences of discrimination among Black people together may exacerbate risk for AD, with variation across geographic contexts. This study examines if discrimination and neighborhood stressors interact to negatively affect cognitive function among Black older adults by region and urbanicity.

**Methods:**

Data are from Black adults >50 years of age (n = 1,690) in the 2010-2016 waves of the Health and Retirement Study. To determine geographic differences in lifetime discrimination (i.e., unfair experiences in housing, employment, and policing), perceived neighborhood stressors (disorder and social discohesion), and cognitive function, descriptive statistics stratified by US region (South versus non-South) and urbanicity (rural versus non-rural) were used. Linear regression models tested the association between cognitive function and two interactions terms: neighborhood disorder*discrimination and neighborhood discohesion*discrimination.

**Results:**

Older Black adults living outside of the South experienced more discrimination events and reported greater neighborhood disorder than those residing in the South, while those living in non-urban areas had lower cognitive function than those in urban areas. Statistically significant interaction terms indicate that among Black adults living in urban areas, greater neighborhood disorder and discohesion along with greater lifetime discrimination was associated with lower cognitive function.

**Discussion:**

Lifetime discrimination may increase Black older adults’ vulnerability for poor cognitive function especially if they reside in stressful, under-resourced neighborhoods. Further exploration of life course factors such as migration and timing of discrimination events will enhance understanding of these findings.

